# Supplementary material for: Differences in the health-related quality of life in patients with asthma living in urban and rural areas in the Amur Region of Russian Federation
Source: PLoS One. 2023 Nov 14;18(11):e0284683. doi: 10.1371/journal.pone.0284683 (PMC10645338; doi:10.1371/journal.pone.0284683)
Supplement: Attachment — Submitted filename: Response to reviewers.docx [file pone.0284683.s001.docx]

**Response to reviewers**

Dear Reviewer #1,

We thank you for your great work in reviewing our manuscript and for your comments. Below are the responses to the comments and information about the corrections made:

- *Introduction section should be shortened. All statements should be followed by corresponding references. All statements without references should be deleted. Exact region where patients live should be mentioned instead of the country name as it was a small regional study.*

Response: In accordance with the comments, we have shortened the "Introduction" section. All statements are followed by corresponding references. All unlinked statements have been deleted. The country name in the title and text of the article is supplemented with a mention of the specific region in which the study was conducted (Amur Region of the Russian Federation).

- *Explanatory data on each of used questionnaire should be provided in methods section. Information of Russian translation and validation of used instruments should also be provided.*

Response: Now the “Materials and Methods” section provides complete information on each of the questionnaires, as well as information on the translation into Russian and validation of the tools used with the appropriate links.

- *All abbreviations should be explained when first mentioned.*

Response: In accordance with the remark, all abbreviations are explained when first mentioned and in the notes under tables and figure.

- *It is unclear how data of rural patients was collected. Was it collected by rural medical authorities?*

Response: We clarify that all patients were examined and interviewed during a consultation appointment at the consultative and diagnostic department of the Far Eastern Scientific Center of Physiology and Pathology of Respiration. Each patient completed the questionnaire independently in the presence of a health worker. Rural medical authorities only sent patients for consultation.

- *Lowe scores of SF-36 mean better QoL. Therefore, it is important each time clearly indicate if authors mean higher or worse QoL or SF-36 scores.*

Response: You are correct in pointing out that higher Lowe scores mean higher QoL. We reflected this in the first sentence of the “Quality of life assessed by SF-36 and AQLQ scores” section (Page 11): “A larger number of points on the SF-36 scales among urban residents corresponded to a higher level of QoL…”

- *Conclusions should be based exclusively on study results.*

Response: We corrected the conclusions in accordance with the results of the study.

- *Tables should be in the same style, all abbreviations should be explained.*

Response: The tables are unified. All abbreviations are explained in the notes under the tables.

- *Minor comment: “with no annual dynamics” – how dynamics was studied if patients filled in questionnaires only once?*

Response: Patients filled out questionnaires once, indeed. The mention in the text about the annual dynamics is not the result of a repeated study, but only an analysis of the answers to the second question of the SF-36 questionnaire: “Compared to one year ago, how would you rate your health in general now?”

Once again, thank you for your positive review and comments.

Sincerely,

Dr. Natalia M. Perelman

Dear Reviewer #2,

We thank you for your great work in reviewing our manuscript and for your comments. Below are the responses to the comments and information about the corrections made:

- *Abstract: Conclusion is not a conclusion, but more a summary. Please write an appropriate conclusion, which is supported by the important outcomes of the study.*

Response: We corrected the conclusions in accordance with the results of the study.

- *Introduction: Page 3, Line 50. The first statement needs rephrasing.*

Response: We have rephrased the statement (Page 3, Lines 55-57).

- *Page 4, Lines 73-75: “Information and intellectual overloads cause ……….. negatively affects the course and prognosis of a chronic disease”. Reference missing.*

Response: We have removed this sentence from the text.

- *Page 4, Lines 75-78: “Geographical remoteness of settlements, poor quality …………. rural communities in the Russian Federation.” How this statement is relevant to the current study?*

Response: We have removed this sentence from the text.

- *Page 4, Lines 78-83: “In addition, rural residents suffer from declining …………… give patients recommendations for a healthy lifestyle and behavior.” The authors did not provide any supporting references in support of these statements.*

Response: We have removed this sentence from the text.

- *Page 4, Lines 94-96: “The purpose of this study is to assess the influence of social factors on ……………… permanently residing in urban and rural areas. What are the social factors that the Authors have considered for the current study? It should be explained here.*

Response: We have made a more correct description of the purpose of this study (Page 4, Lines 82-84).

- *The Introduction section is very vague. It should be focused and precise and should be written keeping in mind the objectives of the study.*

Response: We have shortened and corrected the "Introduction" section.

- *Page 5, Lines 100-101: “The duration of the disease in the 101 sample ranged from 1.5 to 28 years.” The Authors should also have examined the impact of the ‘duration of the disease’ on the QoL. Does ‘the duration of the disease’ similar between urban and rural residents?*

Response: We did not set ourselves the task of studying the effect of “disease duration” on QoL. However, to exclude such speculations, we additionally provided Table 1, in which we showed the same distribution of patients with different duration of illness in groups of urban and rural residents.

- *Spirometry outcome variables should be explained.*

Response: We have added a detailed description of the spirometry outcome variables (Page 7, Lines 130-137).

- *All the questionnaires should be explained in detail, such as the number of items, scoring, interpretation of scores, etc.*

Response: We have added a detailed description of all questionnaires.

- *Statistical analyses: Results of Kolmogorov-Smirnov and Pearson-Mises should have been given that reflect the normality of the data.*

Response: We entered into the tables the values of the Kolmogorov-Smirnov criterion, from which the data normal distribution follows. The Pearson-Mises criterion has been removed from the text, since this criterion duplicates the results of assessing the distribution normality using the Kolmogorov-Smirnov criterion.

- *Table 1: In the column title ‘urban’ and ‘rural’ should have been written in place of ‘1 group’ and ‘2 group.’ Abbreviated variables in the 1st column should be spelled out in the footnote. t-value and p-value should be given in a separate column.*

Response: We have made all the corrections indicated (see Table 2 on Page 10)

- *Correlation results should be given separately in the main text in a separate sub-heading and values should be given in a Table.*

Response: We have separated the correlation results into the subsection “Correlation analysis data” (Page 14), providing it with Tables 6-8, which contain comprehensive information about the correlation coefficients and their significance.

- *Page 11, Lines 181-182: “However, in the urban residents group, the level of “Activity limitation” correlated with all domains of the SF-36 questionnaire.” The authors did not specify whether the correlation is positive or negative.*

Response: We have additionally indicated whether there are positive or negative correlations in all cases.

- *Figure 1 is not clear.*

Response: The figure reflects statistically significant correlations between the domains of the SF-36 and AQLQ questionnaires, as well as the smoking index and ACT. It clearly demonstrates a significant depletion of the field of correlations in rural residents with asthma compared to urban residents. In our opinion, the disappearance of interrelationships of some QoL domains in rural residents suggested a disunity of the physical, psychological and social aspects of life, which negatively affects the QoL level among asthma patients. These explanations are given on Pages 15-16 (Lines 284-289) and Page 22 (Lines 327-332).

- *Page 12, Lines 194-196: “Among the asthma patients living in urban areas, …………. significantly higher than in rural residents (84 vs. 38).” What is 84 vs. 38? The Correlation result is not written properly. Correlation coefficient values are not given.*

Response: We have changed this sentence (Page 16, Lines 287-289). It reflects in text form the above information about a significantly smaller number of statistically significant correlations of QoL domains with each other in rural residents compared to urban residents. 84 significant correlations were found among urban residents, and only 38 among rural residents.

- *Page 12, Lines 204-205: “We did not observe any differences …………… on the HADS.” The authors did not mention the “difference between which group.”*

Response: We explained which groups did not show differences: “We did not observe any differences in the level of anxiety and depression on the HADS between groups of urban and rural residents” (Page 13, lines 229-230)

- *Page 12, Line 206: ‘subclinical level;’ what does it mean?*

*Page 12, Lines 206 and 207: What is the difference between subclinical level and clinical level?*

Response: We have given the necessary explanations in the section “Methods” (subsection “Anxiety and depression assessment”, Page 8, Lines 167-169).

- *Results of HADS should be given in a Table.*

Response: HADS results are listed in a separate Table 5 (Page 13)

- *Pages 12 and 13, Lines 207-209: “The number of urban residents with a ……… rural residents (34.8%; χ²=34.08; p<0.001). What is the frequency of urban and rural residents with clinically significant levels of anxiety?*

Response: We corrected the sentence (Page 13, Lines 236-238): “The frequency of urban residents with a clinically significant level of anxiety (56 persons, or 25.2%) turned out to be lower compared to rural residents (45 person, or 34.8 %; χ²=34.08; p<0.001)

- *Page 13, Lines 209-213: “When analyzing individual 209 questionnaires, we noted that the psycho-emotional background ……………… instability with a predominance of anxiety more frequently.” There is no data/results in support of these statements.*

Response: To support this, we added the data on the frequency of individual responses to the HADS statements “I feel tense or 'wound up'” and “I can sit at ease and feel relaxed” (Page 14, Lines 243-246).

- *Page 13, Lines 215-219: “In the urban residents group, the correlation analysis …………….. correlations of anxiety with the RP, VT, SF, “Activity limitation” domains disappeared.” Again, there is no data/results in support of these statements.*

Response: We moved this statement into a separate subsection “Correlation analysis data” (Page 15, Lines 279-283) and supported it by data in Table 8.

- *All results of the Student's t-test, χ² test, and Pearson correlation analysis should be given in Tables or Figures. Also, the result of Kolmogorov-Smirnov and Pearson-Mises criteria should be described.*

Response: In accordance with the remark, we have given all the results of the Student's test, the χ² test, Pearson's correlation analysis and the Kolmogorov-Smirnov test in tables and in the text of the article. The Pearson-Mises test was removed from the description as duplicating the results of checking the distribution normality using the Kolmogorov-Smirnov test.

- *Abbreviated variables in all the Tables should be spelled out in the footnote.*

Response: All abbreviated variables are explained in notes to tables and figure.

- *Page 16, Lines 286-288: “In our study, the absence of correlations between the smoking index and the QoL in the rural residents suggested their riskier behavior in relation to their own health.” This reviewer is unable to understand the above statement.*

Response: This statement has been rephrased in a more correct form (Page 23, Lines 354-358)

- *Page 16, Lines 294-296: “One of the explanations may lie in the varying impact of environmental factors on rural and urban residents, domestic animal ownership, as well as lower intake of controller medications.” This statement is not clear to me.*

Response: We have replaced this sentence with a more likely explanation (Page 24, Lines 360-362)

- *The conclusions however let it down as this is more of a summary than a conclusion. Please write an appropriate conclusion, which is supported by the important outcomes of the study.*

Response: We agreed with the remark and changed the conclusions.

Once again, we sincerely thank you for your thorough review and comments on the substance of the article.

Sincerely,

Dr. Natalia M. Perelman
