# Supplementary material for: Differences in the health-related quality of life in patients with asthma living in urban and rural areas in the Amur Region of Russian Federation
Source: PLoS One. 2023 Nov 14;18(11):e0284683. doi: 10.1371/journal.pone.0284683 (PMC10645338; doi:10.1371/journal.pone.0284683)
Supplement: Attachment — Submitted filename: Reply to reviewers10022023.docx [file pone.0284683.s002.docx]

**Response to reviewers**

Dear Reviewer #1,

We thank you for your great work in reviewing our manuscript and for your comments. Below are the responses to the comments and information about the corrections made:

- *Authors studied several characteristics of patients with asthma. Why only QoL is mentioned in the title?*

Response: We aimed to study the differences in the QoL of patients with asthma, depending on the living conditions. The remaining characteristics are used to reveal the causes and relationships of these differences. Therefore, the title reflects only the QoL.

- *Presented study limitations are not fully adequate. It was not a real epidemiologic study and can’t represent the entire region. Data was collected from a single city hospital and included only those patients who were treated in that hospital.*

Response: The authors did not claim this study as epidemiological. Based on the recommendations previously put forward by the reviewers, we have already made a clarification on the location of the study. The title and text of the manuscript reflects the specific area (Amur Region) in which the research was carried out. Patients living in different villages and cities of the Amur Region come to our research clinic for a consultation, so the population of the region is widely represented in the sample. However, the limitation mentioned by the reviewer is already included in the text (Page 25, Lines 374-375).

- *Lower number of included rural patients may influence statistical analysis (in particular correlation coefficients in rural patients).*

Response: The difference in the number of rural and urban residents included in the study indeed may influence the absolute values of the correlation coefficients. However, it is taken into account when calculating *p* according to the criterion *t*. The absence of most relationships between QoL indicators in the group of rural respondents was confirmed statistically.

- *Authors should make the language more clear for readers.*

Response: We have tried to present the results of our research as clearly as possible. Claims to the style of manuscript are most often subjective, and it is difficult for us to understand what exactly does not suit the reviewer.

- *The text should be shortened. All unrelated information should be deleted. Authors should present all differences and discuss it in Discussion section of the manuscript.*

Response: The text has been shortened where possible without compromising completeness. Previously, we significantly increased the volume of the text, especially in the Methods section, at the request of the reviewers themselves. Unrelated information has been removed. All differences are presented in the Results section and discussed in the Discussion section.

- *Abstract. "In the urban group, the correlation analysis demonstrated a clear influence of RE on patients’ own assessment of their physical status of QoL” – What is“physical status of QoL”? How scored?*

Response: We replaced the term “physical status” with the more adequate term “physical functioning” and supplemented it with a correlation coefficient between RE and PF (Page 2, Line 38). QoL domains reflect patients' perceptions of the physical, emotional, and social aspects of life. The physical health measure by SF-36 includes physical functioning (PF, ten items), role-physical (RP, four items), bodily pain (BP, two items).

- *Why correlation coefficients presented only in some cases? Correlation coefficients under 0.4 correspond to weak correlation.*

Response: We have added correlation coefficient to the Results in the Abstract (Page 2, Line 38). Unfortunately, the abstract is limited by the requirements of the journal, so the following text has been changed without values of the correlation coefficients. The significance of the correlation coefficient is determined not by the absolute value, which depends on the number of correlation pairs, but by the probability the null hypothesis, which is characterized by the p value. We do not state anywhere that correlation coefficients below 0.4 correspond to a strong correlation.

- *It is difficult to understand the following statement: “The disappearance of interrelationships of some QoL domains in rural residents can be regarded as a separation of the physical, psychological and social aspects of life, which negatively affects the QoL”. It is also unclear why this statement was placed in Results section of the abstract.*

Response: We have changed the description of the Results section in the abstract. But we would like to give an explanation in response to the remark. In a cohort of urban residents, we revealed versatile correlations between different domains of QoL. For example, significant correlation coefficient was between PF and MH. The first indicator reflects the physical functioning, the second - the mental health, fnd both correlate with SF, which reflects social functioning. In the cohort of rural residents, there were no statistically significant correlations between SF, PF and MH. We suggest that the absence of interrelationships in some areas of QoL among rural residents can be considered as a dissociation of patients' perception of the physical, psychological and social aspects of life.

- *Significant difference was reported only for a single SF-36 subscale “Role emotional”. Data presented in conclusion of the abstract is absent in abstract Results.*

Response: We added Results by data on more frequent use of short-acting beta-agonists and higher incidence of clinical level anxiety in rural residents (Page 3, Lines 45-50). Thus, the data presented in the conclusion of the theses began to correspond to the results.

- *It is difficult to understand authors’ idea: “Subjective perception of health and lifestyle by the members of urban and rural communities are risk factors for the development of exacerbations and progression of asthma”.*

Response: We reformulated more correctly (Page 4, Lines 70-71).

- *“…reasons for the existing differences in the course of asthma in urban and rural residents” – Why authors were sure that this difference exists?*

Response: We present in Table 2 data on the greater need for beta-2-agonists in rural patients with asthma. In addition, references are made in the previous reformulated sentence to available literature data. However, here we have corrected to: … existing possible differences … (Page 4, Line 87)

- *“…disappearance of the correlation” – Absence of correlation?*

Response: We corrected to “…absence of the correlation…” (Page 14, Lines 262-263).

- *Authors stated that “the burden of disease had a more significant impact on the QoL of rural residents.” However, presented results showed no difference in asthma-specific HRQoL instrument and difference in a single domain of generic SF-36 that may not be related to asthma.*

Response: We agree that the differences we have identified from the questionnaire data may not be convincing enough for this statement. Taking into account the data of the correlation analysis, which showed the absence of many correlations between various QoL domains in rural residents compared to urban residents, we changed the first sentence in the Conclusions: “The burden of asthma introduces a greater imbalance in the health-related QoL of rural residents compared to urban residents in the Amur region of the Russian Federation” (Page 25, Lines 382-384).

- *What is the base for the following statement: “rural patients with asthma are not able to comprehensively assess their current health and the risks for its deterioration. On the contrary, a relatively high level medical awareness and readiness for health preservation results in a better QoL of urban residents with asthma.”?*

Response: This statement is based on evidence of a lower level of education in the cohort of rural residents in the study (Page 5, Lines 101-104). In addition, the lower level of education, culture and availability of qualified medical care among rural residents in the Russian Federation compared to urban residents is reflected in the source [20]. Similar data are given in [16]: “Overall rural US populations face increased poverty and inferior health care for reasons related to insurance status and to poor access due to increased travel distance and lack of providers, particularly specialists”. We have changed the sentence to express this statement in a less categorical form: “Rural patients with asthma are more often unable comprehensively assess…” (Page 23, Lines 326-327).

Once again, thank you for your review and comments.

Sincerely,

Dr. Natalia M. Perelman

Dear Reviewer #2,

We thank you for your work in reviewing our manuscript and for your comments. Below are the responses to the comments and information about the corrections made:

- *Abstract: Page 6, Lines 116-118 and 119-121: “Dyspnoea was assessed ……. more severe dyspnoea” and “The degree of dyspnea ….. more severe dyspnea.” What is the difference between these two sentences?*

Response: We removed the erroneous duplicate.

- *Tables 2-5: As suggested by the reviewers, the authors did not provide the t-value and p-value in a separate column in the tables.*

Response: We added to the table 2-5 columns with t values.

- *Table 4: The authors did not revise the column title.*

Response: We have corrected the column title in Table 4.

- *Page 15, Lines 267-268: “correlation analysis showed a negative effect of smoking (SI) on GH, PF and RP.” The correlation analysis does not imply causation (cause and effect), it only shows an association between/among the variables. Therefore, rephrase the sentence.*

Response: We have rephrased the sentence (Page 15, Lines 271-272).

- *Tables 6-8: The authors should write the values of correlation coefficients and p-values instead of writing ‘NS,’ although the relationship is not significant.*

Response: We have written the correlation coefficients and p values in Table 6-8.

Once again, we sincerely thank you for your review and comments.

Sincerely,

Dr. Natalia M. Perelman
